# Book Review: Man's Search for Meaning (Victor Frankl)

**DOI:** 10.3389/fpsyg.2016.01493

**Published:** 2016-11-16

**Authors:** Khushali Adhiya-Shah

**Affiliations:** Department of Psychology, Mithibai College of ArtsMumbai, India

**Keywords:** search for meaning, Victor Frankl, meaningfulness, suffering, man's search for meaning, depression, anticipatory anxieties

Humans in suffering tend to feel hopeless with a deep sense of failure. MAN's SEARCH FOR MEANING (Frankl, 1984) is a helpful book during such times: it is highly probable that one would find a solution to their depressed feelings, if the book is read actively.

Written by Austrian neurologist-psychiatrist and a Holocaust survivor Victor Frankl, this book is simple yet intense and reflective. Frankl is the founder of Logotherapy, a form of existential psychology. Awarded with several accolades, his books and talks are the most inspiring on finding meaning in life and in suffering.

The book stands out extraordinarily as Frankl, by narrating his life instances in the Auschwitz concentration camp, presents the remarkable idea of how we can choose to see a purpose or meaning in any situation, including the worst conditions. He descriptively illustrates his personal experiences and observations of minute human changes which infuses hope into the reader.

With rich primary and secondary data, Frankl puts forward his ideas in three sections. The qualitative methodology utilized has smoothly fused his thoughts through these three parts, clarifying Nietzsche words, “He who has a *why* to live can bear almost any *how*.” This book is a collaboration of Frankl's personal experiences and stories, references to other existential forerunners, quotes from humanistic and psychoanalytic schools, and excellent figurative examples. Many pathological terms have been used in the book, which are well explained by the author.

The first section describes the brutality every prisoner faced at concentration camps, Frankl being one of them for three years. As he realized their “naked existence,” Frankl begins by explaining how a prisoner passes through three major phases in the camp, and also how each phase transformed the prisoners from their previous lives and how they developed various pathologies. The prisoner was first in a state of shock, which was followed by the phase of developing apathy and finally, on being liberated, prisoners felt depersonalized at first and later manifested strong symptoms in differential ways.

Frankl here slowly introduces his first thoughts on these experiences. Though he has toned down the language of brutality, the message comes across loud that it was certainly the worst suffering one could imagine of.

At the end of section one, an active reader realizes the true meaning of life, of love (which is fairly depersonalized in the recent decades) and also how thankless we have become toward the little mercies in life.

An active reader also learns about “Logotherapy” that the author attempts to explain in the second section. The nature, meaning and goals are well detailed. Even the finest differences between psychoanalysis and Logotherapy are clearly specified. Frankl liberally introduces every concept of Logotherapy (such as the existential vacuum, responsibility of survival, existential frustration). He also describes the therapy process and techniques with some great figurative examples and case studies. A novice therapist may find these useful. However, he fails to explain how one can integrate these techniques with the conventional psychotherapeutic process.

Nevertheless, his strong request to re-humanize psychotherapy inspires us into a new direction of thought and practice.

The third aspect of the book is an attraction for readers wishing to apply the principles of Logotherapy on the self (to begin with): the section on tragic optimism elaborates it. The triad of pain, guilt and death is well justified, though further intensive reading is necessary for a practicing therapist.

This section is also useful for a therapist to understand how anticipatory anxieties, depression, obsessive behaviors, aggression, unemployment neurosis (and even Sunday neurosis) can be dealt with effectively through Logotherapy. Frankl takes the effort to explain how meaninglessness in life may not be pathological, but can certainly be pathogenic. However, this section is exhaustive to comprehend with the given information, and hence, would suggest further reading of the ‘Tragic Triad’ for a practitioner.

Having justified the idea of finding meaning in life, this book extends itself to coherently explain where and how one can find their purpose in life—reading this section of the book will most certainly spark a solution to every despaired reader. Frankl positively disregards a specific age group that can benefit from this book because he elucidates how old age and death must not be looked upon as an “end of opportunities and possibilities,” but as a repertoire of all the “potentials actualized, meanings fulfilled and values realized.”

He also explains how “suffering is not necessary to find meaning.” If suffering can be avoided, meaningfulness would lie in attacking the cause of suffering; but if it can't, meaningfulness would lie in changing the way we look at the situation and unlock the actual meaning lying “dormant” in that suffering! This relates very well to the “*Serenity Prayer*.”

Readers having knowledge in the Indian philosophy may easily connect the ideas of this book to the *Bhagavad-Gita* (Bhaktivedanta Swami Prabhupada, 1978) wherein Lord Krishna explains to Arjuna (and the humankind thereby) about how he could find meaning in his dreadful situation, how his suffering may be looked upon differently and how one can elevate oneself from hopelessness and anguish by realizing the purpose of one's existence on earth.

Clinically as a limitation, the book lacks presentation of validity, procedure and practice of Logotherapy. The therapy doesn't easily allow a quantitative inquiry: it is a philosophical approach to the human inner-world (as Frankl describes it). Despite the shortcomings, the spirit of the idea is noteworthy.

Recent researches have also well supported Frankl's ideas. Thagard (2012) in *The Brain and The Meaning of Life*, argues how brain science matters for fundamental issues about meaning in life.

Positive correlations have been found between search for meaning with other variables such as positive affect (King et al., 2006), well-being (Mascaro and Rosen, 2005), and self-evaluation (To et al., 2014). Steger et al. (2008) found that people lacked the search for meaning in life through a life-span perspective (Steger et al., 2007).

Ubiquitously, I would strongly recommend this book as the first step into existential psychology and urge the reader to continue reading Frankl's other books.

## Author contributions

The author confirms being the sole contributor of this work and approved it for publication.

### Conflict of interest statement

The author declares that the research was conducted in the absence of any commercial or financial relationships that could be construed as a potential conflict of interest.
